# Oocyte Cryostorage to Preserve Fertility in Oncological Patients

**DOI:** 10.1155/2012/525896

**Published:** 2012-01-15

**Authors:** Alberto Revelli, Emanuela Molinari, Francesca Salvagno, Luisa Delle Piane, Elisabetta Dolfin, Simona Ochetti

**Affiliations:** Phisiopathology of Reproduction and IVF Unit, Department of Ginecological and Obstetrical Sciences, S. Anna Hospital, University of Torino, 10126 Torino, Italy

## Abstract

Thanks to the progress in oncostatic treatments, young women affected by cancer have a fairly good chance of surviving the disease and leading a normal post-cancer life. Quite often, however, polychemiotherapy and/or radiotherapy can induce ovarian damage and significantly reduce the content of follicles and oocytes inside the ovary, thus predisposing the patient to menstrual disorders, infertility, and precocious menopause. Several techniques have been proposed to preserve fertility in these patients; among them oocyte collection and cryopreservation prior to the oncostatic treatment has been widely applied in the last decade. The proper indications, the permitting conditions, the available hormonal stimulation protocols, as well as the effectiveness and limits of this option will be discussed herein, with a comprehensive and up-to-date review of the two techniques commonly used to cryostore oocytes, the slow-freezing technique and the vitrification technique.

## 1. Introduction

During the past decade, fertility preservation has become an important issue in cancer patients' management. Survival rates after malignancy treatment have improved markedly, especially for young women affected by melanoma, oncohaematological diseases, and breast cancer, leading to the generation of long-term cancer survivors [1, 2]; nonetheless, this population is quite frequently affected by iatrogenic infertility and/or premature ovarian insufficiency (POI) [3]. Recent studies, in fact, showed that abdominal radiotherapy may lead to ovarian damage in a dose-dependent fashion; similarly, total body irradiation may result in POI in about 97% of cases [4]. Chemotherapy regimens, especially those involving alkylating agents, may cause acute loss of follicles within the ovary, leading to hormone deficiency and permanent infertility [5]. Psychological distress induced by the loss of reproductive perspective as well as factors related to a premature menopause (osteoporosis, cardiovascular diseases, depression, etc.) may dramatically affect survived women's quality of life. Although disease remission obviously remains the first goal of cancer treatment, patient's awareness toward safeguarding future fertility is increasing [6]. 

Many approaches have been considered to preserve fertility and avoid POI. Embryo cryostorage has been considered for years the only valid option, albeit this procedure is applicable only to stable couples and not to singles, and still raises a lot of ethical, legal, and practical concerns. Ovarian cortex ablation and cryostorage, with subsequent autografting, is the only strategy which can be proposed to prepubertal girls, though it is still considered an experimental approach with limited results.

Nowadays, oocyte cryostorage is considered an important tool for fertility preservation worldwide, as no surgery is required and minimally invasive ovarian stimulation protocols are available. Moreover, storing oocytes implies no concerns regarding possible cancer cells contamination, a problem that affects ovarian cortex retransplantation strategy [7]. As a result of oocyte cryostorage and *in vitro* fertilization (IVF), over 2000 babies were born in nononcological, routine IVF patients.

## 2. History of Oocyte Cryostorage

Since oocyte cryostorage was introduced in the mid 1980s, general interest rose around the possibility of increasing pregnancy rates using frozen eggs, overcoming the ethical and legal concerns related to embryo freezing.

Since the very beginning oocyte freezing was quite problematic, with survival and fertility rates below 50%, and pregnancy rates as low as 1-2% [8–10]. Chen and Van Uem et al. reported the first pregnancies obtained after oocyte freezing/thawing, raising many expectations in scientific community [11, 12]. Unfortunately, many other attempts failed to reach the same result [13–15]: oocyte freezing strategy was dramatically less effective than zygote or embryo cryopreservation. Before intracytoplasmatic sperm injection (ICSI) was introduced in most IVF laboratories, the premature release of cortical granules by the frozen oocyte with the consequent irreversible thickening of the zona pellucida was able to halt sperm penetration and impair fertilization [16, 17].

Cryobiologists encountered several problems in freezing oocytes, including ice crystal formation, osmotic stress, and cryoprotectant agents (CPAs) toxicity [8, 18, 19]. Compared to other mammalian cells, human mature oocytes are constituted by a very high amount of water and have a small surface-to-volume ratio, which strongly affects cells dehydration that is essential for survival after thawing. Zona pellucida cracking, mitochondria shrinkage, and microfilaments alteration were also addressed as cryostorage-induced injuries on the human oocyte [20]. Moreover, meiotic spindle (MS) disassembly induced by cooling was clearly shown after the introduction of polarized light microscopy analysis. Many authors reported that when oocytes are exposed to low temperatures, the MS disappears from their oocytes, and reappears as a consequence of repolymerization after a few hours of incubation at thawing temperature [21, 22]. The survival of an oocyte after warming can be assessed when a bright cytoplasm surrounded by an intact zona pellucida is observed; anyway, chilling female gametes to subzero temperatures provides damages to their ultrastructure, as observed by several authors who performed electron microscope analysis: the main consequences of freezing/thawing procedures involve organelle displacement, mitochondrial disruption, vacuolization of the ooplasm, and loss of spindle polarity predisposing to an altered chromosomal alignment [23–25].

Cryobiology aims at minimizing these harmful effects on the human oocyte, and by now, two well-established laboratory protocols have been proposed and are widely diffused in the clinical practice: the slow freezing protocol and the vitrification procedure.

## 3. Candidates for Oocyte Cryopreservation

Fertility preservation should be discussed with all young women at high risk of POI. The most common cause of POI are ovarotoxic anticancer therapies, and cancer patients are by far the main candidates to fertility preservation. However, benign diseases like some genetic syndromes (Turner's syndrome, X-fragile carrier condition, etc.), ovarian diseases (severe endometriosis or ovarian cysts requiring ablative surgery), or autoimmune disorders requiring immunosuppressive therapy may determine POI as well.

Cancer in women in reproductive age is an increasing problem. The American Cancer Society estimates that 1/47 women will develop a cancer before the age of 39 (American Cancer Society, Inc., Surveillance Research 2011). Every year, about 200.000 new cases of breast cancer are diagnosed in USA, 15% of which occur in women under the age of 45. Most of these patients will receive adjuvant chemotherapy with alkylating drugs 4 to 6 weeks after surgery, developing a high risk of POI [26] (National Institute of Health Consensus Development Panel, 2001). Many breast cancer cases, however, are hormone-sensitive, and appropriate ovarian stimulation regimens are needed to keep low circulating estrogen levels while stimulating the ovary to cryostore oocytes [27–30]. Haematological malignancies such as lymphoma and leukemia show now quite good survival rates, and the attention is shifting toward the prevention of side effects like infertility [31].

Even nonneoplastic diseases (autoimmune disorders or benign haematologic diseases being treated with chemo- or radiotherapy) should routinely implicate the offer of oocyte cryopreservation [32, 33].

The selection of candidates for fertility preservation is crucial in order to offer the best suitable technique for each patient. Oocyte cryopreservation is probably the best technique to preserve fertility of women without an established partner or wishing to avoid ethical and legal problems associated with embryo cryostorage. When feasible, oocyte cryostorage may be preferable to ovarian tissue freezing because it does not require surgery and has already resulted in many live births [9]. 

The most important limiting factor for oocyte cryopreservation is age: storing oocytes in women after 40 years of age will probably result in a very poor chance to get a pregnancy in the future. Indeed, patients with a poor ovarian reserve have to be screened carefully, since they may not benefit from the treatment. Ovarian reserve assessment is crucial for patients requiring oocyte cryostorage: besides age, the antral follicle count and hormonal parameters such as FSH and anti-Mullerian hormone (AMH) have to be carefully considered. On the other side, oocyte cryopreservation is unavailable for prepubertal girls because the ovarian stimulation used to obtain oocytes needs the full maturity of the hypothalamus-pituitary-ovarian axis.

Another limiting issue is the timing of the procedure: oocyte cryopreservation requires an average of 12 days for ovarian stimulation and ovum pickup (OPU) to occur [34]. Women starting oncostatic therapy in a very short time from diagnosis or having already started chemotherapy may not benefit from oocyte cryostorage.

Oocyte cryopreservation implies a controlled ovarian hyperstimulation with exogenous gonadotropins that leads to largely supraphysiological levels of serum estradiol. Type and stage of the neoplastic disease and the patient's overall health influence the feasibility of an ovarian stimulation, and stimulation protocols must be individualized for every woman. In this perspective, the use of conventional ovarian stimulation protocols is possible only in women with estrogen-insensitive tumours, while hormone-responsive diseases require appropriate regimens [35].

## 4. Ovarian Stimulation Protocol

Each protocol that aims at obtaining oocytes for cryostorage should be (i) safe, with limited risk of stimulating the growth of a pre-existing neoplasia, (ii) fast, with very limited delay in starting cancer therapy, (iii) effective, with good chances of oocyte retrieval.

Ovarian stimulation requires approximately 10–14 days to achieve mature oocytes at OPU. In case of estrogen-sensitive diseases, the protocol with letrozole (5 mg/day from the second day of menstrual cycle for 5–7 days) plus gonadotropins (150–300 IU of recombinant FSH) and GnRH-antagonists [30] is one of the most recommendable: this regimen allows an acceptable oocyte yield and keeps the circulating estradiol levels rather low [36], a GnRH-agonist may be used to trigger the follicle final maturation, minimizing the risk of ovarian hyperstimulation syndrome (OHSS); moreover, letrozole or GnRH-antagonist can be readministered from the day of OPU until blood estradiol concentration falls below 50 pg/mL. In case of shortage of time, alternative regimens include to start stimulation in the luteal phase. Some women, in fact, need an urgent cancer treatment and cannot delay the beginning of the oncostatic therapies until the onset of menstruation; in this case, a GnRH-antagonist is administered to induce an abrupt luteolysis and then gonadotropins are started [37]. The egg retrieval rate is similar to the one observed using longer, conventional stimulation regimens.

A growing literature showing encouraging results of oocyte *in vitro* maturation (IVM) followed by vitrification for cryostorage is now available [38, 39]. This option consists in the possibility to retrieve immature oocytes from unstimulated preantral follicles, which are arrested in the prophase of the first meiotic division. Immature oocyte retrieval followed by *in vitro* maturation (IVM) resulted in several live births [40] and it is claimed that live births could be achieved combining oocyte IVM and vitrification. This technique is safe and theorically effective for all oncological patients, as no hormonal stimulation is needed, and it can be performed with no time restrictions [39]. The effectiveness of the procedure appears to be higher when immature oocytes are first matured *in vitro* and then frozen [41]. The potential of oocyte maturation is, in fact, reduced after vitrification [42, 43]. 

Overall, some data suggest that immature oocytes could be less sensible to cryodamage than mature oocytes because their nuclear apparatus is still not fully developed, and after thawing, they can be matured *in vitro* to metaphase II [56, 57]. Cryopreservation of immature oocytes should be considered in oncological patients who cannot undergo hormonal stimulation with high peak estradiol concentrations, for example, patients with breast cancer [58].

## 5. Slow Freezing Method

The slow freezing/rapid thawing method was the first cryostorage protocol adopted for oocytes in IVF laboratories. It was originally introduced with the aim to preserve supernumerary embryos obtained from assisted reproduction procedures [59, 60].

Oocyte freezing was initially a damping technique: rates of survival and cleavage after thawing were significantly lower than those obtained using zygote or cleavage stage embryos. The major burden of mammalian egg cryopreservation was found to be membrane permeability to cryoprotectants: after fertilization, in the zygote and in the cleavage-stage embryo, water permeability kinetics change, rendering the cells more prone to freezing [61].

The original protocol introduced for mouse embryo cryopreservation was slightly modified and adapted to human cells [62]. Small permeating molecules, like dimethyl-sulfoxide (DMSO) or propandiol (PROH), were adopted to allow water substitution in the intracellular compartment and were found to be useful to avoid ice crystal formation within the oocyte's cytoplasm. Sucrose-supplemented media were effective in reducing the shrinking/swelling phenomenon occurring when osmotic imbalance between the intracellular compartment and the extracellular environment is generated. Some authors [63] observed that increasing sucrose concentration from 0.1 to 0.2 M increased oocyte survival and fertilization after thawing; further raising sucrose concentration up to 0.3 M yielded even better results [64]. Sodium replacement with choline in the cryopreservation medium also obtained satisfactory results [65, 66].

Another crucial point of the oocyte freezing technique is the rate of freezing, which has to be performed under strictly controlled conditions: room temperature, as well as equilibration temperature of cryopreservation media, is able to interfere with membrane permeability to cryoprotectants, possibly affecting the oocyte survival chance [19]. Moreover, since slow freezing technique slowly dehydrates oocyte cytoplasm, a programmable freezer is required in the laboratory. The cooling rate must reach, starting from room temperature (20°C), a temperature of −7/8°C with a speed of −2°C/min. In order to prevent spontaneous ice crystal formation, at this stage the operator must perform manual seeding by touching the device where oocytes have been previously loaded (usually a plastic straw) with a nitrogen-cooled object. Subsequently, samples are cooled to −30°C at a speed rate of −0,3°C/min and then definitively frozen to −150°C at a speed rate of −50°C/min. Differently, the warming rate must be very rapid in order to prevent the recrystallization of intracellular water.

The slow freezing protocol has been considered the gold standard technique for oocyte cryopreservation for years; it is a well-established procedure with survival rates usually as high as 60–80% (Table 1) [45, 47, 48, 67, 68]. Nevertheless, some authors emphasized the detrimental effects of high sucrose concentration on oocyte cytoplasm organelles and proposed alternative freezing techniques and timing schedules [23, 52, 69].

Clinical reports on slow freezing show a pregnancy rate ranging between 13 and 20% (pregnancy/embryo transfer) (Table 1) and implantation rates still low in comparison to those observed in fresh cycles [44]. Grifo and Noyes compared slow freezing to vitrification on sibling oocytes, showing similar results in terms of survival, but higher fertilization and blastocyst formation rates using the former [67].

In standard IVF procedures, cryopreserving oocytes combines the chances to achieve a pregnancy by both fresh and thawing cycles, thus yielding a rather high cumulative pregnancy rate [47].

## 6. Vitrification Method

Early studies on oocyte vitrification were performed at the end of 1980s, when the first attempts on mouse or hamster eggs were reported [70, 71]. The introduction of oocyte vitrification in human IVF is a relatively recent phenomenon [46, 54, 72]. 

The scientific basis of vitrification consists in the ultrarapid freezing of cells, whose intra- and extracellular aqueous environment turns into a glassy-like state. Vitrification combines two different biophysical processes: a preliminary equilibration step, in which oocytes are exposed to low concentrations of cryoprotectants to allow water outflow, and a subsequent vitrification phase in which cells undergo a high osmotic gradient that completes cells dehydration. Under these conditions, oocytes can be directly merged into liquid nitrogen and then stored. Similarly, warming of oocytes must be rapid in order to avoid recrystallization of water.

The cryoprotectants used during vitrification are the same employed for slow freezing, but they are three-to-four-folds more concentrated in vitrificaton than in slow freezing. DMSO, PROH, and ethylene glycol (EG) (5-6 M) as well as sucrose (1 M) are currently used, though their toxicity is still under evaluation [73]. 

Appropriate carriers for freezing oocytes are also very important. Successful vitrification occurs when samples are loaded in a minimal fluid volume and then frozen/thawed at an extremely fast rate (1500–2000°C/min). Open systems guarantee direct contact with liquid nitrogen [74, 75]: open-pulled straws, cryo-tops and cryo-loops, cryo-leafs, electron microscopy grids, and many other devices were tested in the last years [39, 46, 76, 77]. All open systems raise some concerns about the possible viral contamination of stored material, either from nitrogen or from cross-contamination among samples [73]: strategies to avoid this risk include the formulation of high-security closed devices, exposure to nitrogen vapors, and nitrogen ultraviolet (UV) sterilization [55, 78].

Although no cross-contamination between liquid nitrogen and stored oocytes has been signalled to date, closed systems may provide the safer and probably most effective vitrification procedure. In particular, many carriers have been approved by FDA in the last years, and several of them are now commercially available: Cryotip (Irvine Scientific, CA, USA), high-security vitrification (HSV) straw (Cryo BioSystem, Paris, France), VitriSafe (VitriMed, Austria), Cryopette (Origio, Denmark), and Rapid-i (Vitrolife Sweden AB) [79]. DNA integrity assessed on warmed mouse oocytes is comparable in open versus closed vitrification systems; anyway, there is still an ongoing debate whether closed or open vitrification carriers provide the best results in terms of fertilization and cleavage rates [80]. On the other hand, there is wide agreement in considering vitrification an operator-dependent procedure.

 Oocyte survival after vitrification reaches 90% in several reports (Table 1) [38, 42, 46, 49, 50, 54, 81]. Oocyte quality seems to be poorly affected by chilling injury: spindle repolymerization occurs within one hour after warming, suggesting that the ultrastructure of these gametes is better preserved by vitrification rather than slow freezing [82]; moreover, the metabolomic profiling of vitrified oocytes was found to be comparable to the one of fresh eggs [83, 84].

Data on the clinical use of vitrified eggs in routine IVF show that pregnancy rates can be comparable to those achieved with fresh oocytes (Table 1) [9, 54, 85]. Studies aimed to compare vitrification and slow freezing reported implantation and pregnancy rates trendly higher with vitrification, although the number of observed cases overall is still too low to draw final conclusions [42, 51, 53, 81, 86].

## 7. Conclusions

Cryostoring oocytes is an effective method to preserve fertility in postpubertal young women at risk of POI. In the last years, significant improvements in the clinical effectiveness of oocyte freezing/thawing techniques have been achieved using both slow freezing method and vitrification. The available trials comparing these two different approaches are still insufficient to establish the superiority of one over the other, but the growing interest of scientist and the increasing awareness of women about the possibility of storing oocytes will likely lead to the development of an optimal protocol for oocyte storage in the next few years. 

## Figures and Tables

**Table 1:** Results from different oocyte cryopreservation protocols: slow freezing (high-sucrose concentration) and vitrification.

	Vitrification (VIT)	Slow freezing (SF)	Survival	Fertilization	Pregnancy	Miscarriage	Egg donation program
Chen et al., 2005 [44]	—	Yes	75% (119)	67% (80)	33% (7)	0%	Partially
Li et al., 2005 [45]	—	Yes	90% (73/81)	82% (60/73)	47% (7/15)	28% (2/7)	Partially
Kuwayama et al., 2005 [46]	Yes	—	91% (58/64)	81% (52/64)	41% (12/29)	17% (2/12)	No
Borini et al., 2006 [47]	—	Yes	43,4% (306/705)	51,6% (158/306)	19,2% (14/73)	28,6% (4/14)	No
Barritt et al., 2007 [48]	—	Yes	86,1% (68/79)	89,7% (61/68)	75% (3/4)	NS	Yes
Lucena et al., 2006 [49]	Yes	—	96,7% (143)	87,2% (105)	56,5% (13)	NS	Yes
Antinori et al., 2007 [50]	Yes	—	99,4% (328/330)	92,9% (305/328)	32,5% (39/120)	20,5% (8/39)	No
Cobo et al., 2008 [51]	Yes	—	96,9% (224/231)	76,3% (171/224)	65,2% (15/23)	20% (3/15)	Yes
Parmegiani et al., 2008 [52]	—	Yes	75,1% (328/437)	80% (227/328)	19% (16/83)	31,2% (5/16)	No
Cao et al., 2009 [42]	Yes	Yes	SF 61% (75/123)	SF 61,3% (46/75)	ND	ND	No
			VIT 91,8% (268/292)	VIT 67,9% (182/268)	ND	ND	No
Smith et al., 2010 [53]	Yes	Yes	SF 65% (155/238)	SF 67% (104/155)	SF 13% (4/30)	SF 25% (1/4)	No
			VIT 75% (260/349)	VIT 77% (200/260)	VIT 38% (18/48)	VIT 18% (4/18)	No
Rienzi et al., 2010 [54]	Yes	—	97% (120/124)	79,2% (95/120)	30,8% (15/39)	20% (3/15)	No
Cobo et al., 2010 [55]	Yes	—	92,5% (3039)	73,3% (NS)	55,4% (148)	NS	Yes

NS = Data not reported.

ND = Data not calculated, not a study endpoint.
